# Adaptation of a Modified Diet Quality Index to Quantify Healthfulness of Food-Related Toy Sets

**DOI:** 10.1089/chi.2021.0273

**Published:** 2022-08-29

**Authors:** Jacqueline R. Poston, Rachel E. Watkins, Stephanie Jilcott Pitts, Virginia C. Stage, Suzanne Lazorick

**Affiliations:** ^1^ Brody School of Medicine, East Carolina University, Greenville, NC, USA.; ^2^ Honors College, East Carolina University, Greenville, NC, USA.; ^3^ Department of Public Health and Brody School of Medicine, East Carolina University, Greenville, NC, USA.; ^4^ Department of Nutrition Science, College of Allied Health Sciences, East Carolina University, Greenville, NC, USA.; ^5^ Department of Pediatrics, Brody School of Medicine, East Carolina University, Greenville, NC, USA.

**Keywords:** child nutrition, diet quality, pretend play, food-related toys, young children

## Abstract

The objective of this cross-sectional study was to examine the construct validity of an adapted modified Diet Quality Index (*a*DQI) as a measure of the healthfulness of food-related toy sets for young children (3–8 years). A standardized online search was used to identify toy sets (*n* = 50) from 10 retailers. An *a*DQI score (*a*DQI score, range 0–50) was determined for each toy set, mean (standard deviation) = 28.7 (6.1). Regression analyses demonstrated a positive association between *a*DQI score and percentage of dairy, refined grains, protein, vegetables, and fruit and inverse association with percentage of desserts, sugar-sweetened beverages, and total number of servings. Sets contained more protein and fewer fruits than recommended. The *a*DQI score demonstrates construct validity to objectively assess the healthfulness of food-related toy sets. There is opportunity for toy manufacturers to make changes to improve the healthfulness in toy sets for young children, and future research can explore the impact of food-related toy sets on nutrition behaviors.

## Introduction

Child psychologists and development theorists agree that pretend play is necessary to build various skills of development, including creativity, problem-solving, cognitive, and social skills.^1,2^ Pretend play begins universally around 2 years of age and is a form of play involving imagination and creation often utilizing props, such as toy kitchens and food.^2^ Food-related toy sets are often seen in settings such as preschools and child care facilities. However, limited research is available to describe the healthfulness of food-related toy sets, and whether or not the nutritional content of these food-related toy sets aligns with dietary recommendations from the USDA.^3^

Previous literature demonstrates pretend play with food-related toy sets often involves activities such as meal planning, food preparation, table preparation, serving food, eating, and cleaning.^4^ Previous literature also suggests preschool-aged children rely primarily on physical characteristics such as color, shape, and texture to classify and interpret their food choices when interacting with food-related toy sets.^4^ However, the nutritional quality represented by food-related toy sets currently on the market is unknown, and it is unclear whether these toy sets support or hinder positive messaging about healthy eating among young children.

Although we do not fully understand the influences food-related toy sets may have on children's pretend play and ultimately their dietary behaviors, the first step in deepening our understanding is developing a tool capable of measuring the healthfulness of the food-related toys currently on the market. Therefore, the purposes of this study were to examine (1) the construct validity of an adapted modified Diet Quality Index (*a*DQI) as a measure of the healthfulness of food-related toy sets for young children (3–8 years) and (2) the healthfulness of food-related toy sets.

## Methods

For this cross-sectional descriptive study, 10 online well-known retailers of toys (Amazon, Pottery Barn, etc.) were de-identified as “A”-“J” and used for toy selection. In December 2019, retailer sites were searched using standard procedures to find food-related toy sets intended for pretend play for young children. Products included contained sets of toy foods or items intended to create a meal or meals. Lists of up to 13 sets were made and ranked by order of appearance for each retailer, of which the first 5 unique sets containing more than 1 food group were included for the final 50 sets in the study. Of note, 19 of the 25 sets excluded for containing 1 food group contained only desserts. Duplicates in the top 5 of different retailers were only scored once. However, duplicates were documented for inclusion in secondary analyses comparing the top 5 by retailer.

Once selected, toy set contents were analyzed to determine the type and quantity (number of servings or portions) of foods, beverages, and meal preparation items included. FoodData Central^3^ and MyPlate.gov^5^ informed assignment of serving size. Independent variables included retailer, price, total number of items, number of scored items, number of food categories included, whether food preparation items were included (yes/no), number of food preparation items (if applicable), total number of food servings, and number and percentage of total servings of each food category (Example in Supplementary Table S1).

To evaluate the nutritional content, we utilized the modified DQI, a tool used in US national studies to quantify healthfulness of a child's dietary intake by scoring food and nutrients based on alignment with dietary guidelines.^6^ Diet quality indices are increasingly being used to examine associations between dietary intake and health outcomes^7^ or to assess the food environment.^8^ In this study, we adapted the modified Diet Quality Index Score for young children to score each toy set.^9^ The modified DQI Score includes 10 food group categories with a score ranging from 0 to 45 points (higher indicating better nutrition). Eight were scored from 0 to 5 points (dairy, proteins, vegetables, fruits, 100% fruit juices, sugar-sweetened beverages, other added sugars, and salty snacks); two groups were scored from 0 to 2.5 points (whole and refined grains).^9^

We adapted the modified DQI by renaming the “other added sugars” category to “Desserts, treats and other added sugars,” and scoring it as 10 points for 0 servings, 5 for 1 serving, and 0 for ≥2 servings; for a total possible *a*DQI score of 50. Recommended portions were those recommended for 4-year-old children.^10–13^ Two researchers independently scored each toy set; any discrepancies were resolved to determine the final score. Of note, grains were “refined” unless explicitly listed as a whole grain. Juice was considered sugar-sweetened beverages unless explicitly labeled as 100% fruit juice. Foods packaged for multiperson use (*e.g.,* cereal box) were standardized as one portion. Whole fruits and vegetables were counted as one serving.

IRB approval was not obtained because there were no human subjects.

### Data Analysis

The primary outcome variable was the *a*DQI score, with a higher score indicating a more healthful toy set. Descriptive statistics (means, standard deviations, and medians) were computed. To examine factors associated with the *a*DQI score, we calculated the Spearman correlation coefficients and used the backward selection method to determine the multiple linear regression model. Potential explanatory variables included price, number of tools, number of items, percentages of dairy, refined grains, proteins, vegetables, fruit, desserts, sugar-sweetened beverages, and total number of servings. We dropped two toy sets from the analysis because the prices were outliers (>$150.00). We dropped the whole grain variable from the analyses due to low frequency (2/48). We used nonparametric Kruskal–Wallis tests to examine differences by retailer (the top 5 toy sets at each of the 10 retailers with duplicates included). Analyses were conducted using SAS 9.4 (SAS Institutes, Cary, North Carolina, 2013). Statistical significance level was set at alpha = 0.05.

## Results

The characteristics of the toy sets and the *a*DQI scores are shown in Table 1. The mean (range) *a*DQI score was 29 (15.0–37.5), price $25.46 ($10.99–$49.99). The average percent of total servings by food group demonstrated a high prevalence of protein (32%) and refined grains (22%), higher percent vegetables than fruits (19% versus 7%), low percent of desserts (7%), and very low percent sugar-sweetened beverages (0.94%) and whole grains (0.17%) (Supplementary Fig. S1).

**Table 1.** Characteristics of the Toy Sets and Components of the Adapted Modified Diet Quality Index Score: Means, Standard Deviations, Medians, and Ranges (*N* = 48 Toy Sets)

Variables	Mean	Standard deviation	Median	Range
Adapted modified diet quality index score	28.7	6.1	29.4	15.0–37.5
Price	$25.46	7.77	24.99	$10.99–$49.99
Number of tools	2.0	2.3	1.0	0.0–10.0
Number of items	31.2	26.4	20.5	5.0–122.0
Percent diary	9.1	13.1	4.5	0.0–64.0
Percent whole grain	0.2	1.2	0.0	0.0–15.1
Percent refined grain	21.9	15.1	20.5	0.0–57.0
Percent protein	32.0	23.4	29.0	0.0–81.0
Percent vegetable	18.6	19.2	14.0	0.0–98.0
Percent fruit	7.7	10.5	1.0	0.0–40.0
Percent dessert	7.0	12.3	0.0	0.0–64.0
Percent sugar-sweetened beverage	0.9	2.5	0.0	0.0–11.0
Total number of servings	21.1	21.9	13.6	2.8–109.3

There was an inverse correlation between *a*DQI score and percentage of desserts (*r* = −0.76, *p* < 0.0001) and sugar-sweetened beverages (*r* = −0.39, *p* = 0.0063), and a positive association for vegetables (*r* = 0.40, *p* = 0.0048). There was no significant difference in *a*DQI score by retailer (Supplementary Fig. S2). Regression analyses demonstrated a positive association between *a*DQI score and percentage of dairy, refined grains, protein, vegetables, and fruit; and an inverse association for desserts, sugar-sweetened beverages, and total servings. Parameter estimates, standard errors, and *p*-values are included in Table 2.

**Table 2.** Parameter Estimates, Standard Errors, and *p*-Values for Variables Included in the Linear Regression Model of Association with the Adapted Modified Diet Quality Index Score

Predictor	Parameter estimate	Standard error	*p*
Percentage of dairy	0.18509	0.05249	0.001
Percentage of refined grains	0.12629	0.04720	0.01
Percentage of proteins	0.11586	0.04454	0.01
Percentage of vegetables	0.16327	0.04573	0.001
Percentage of fruits	0.27818	0.05806	<0.001
Percentage of desserts	–0.13374	0.06448	0.04
Percentage of sugar-sweetened beverages	–0.44296	0.18193	0.02
Total number of servings	–0.13476	0.01974	<0.001

## Discussion

This study aimed to assess the use of an adapted Diet Quality Index to measure the healthfulness and alignment with suggested nutritional recommendations of food-related toy sets available to young children. Results showed that generally the food-related toy sets did not include sugar-sweetened beverages and whole grains but did include protein, vegetables, and refined grains. When compared with dietary recommendations, the average sets contained fewer fruits and far more protein than recommended. The amounts of vegetables and grains in the toy sets were similar to federal recommendations.^3^ When comparing what young children consume, prior literature demonstrates children are not consuming as many fruits, vegetables, whole grains, or protein as recommended.^14^

There was no significant difference in *a*DQI scores between retailers. Toys with higher *a*DQI scores, representing more healthful nutritional content, were more likely to contain protein, dairy, refined grains, vegetables, and fruit; and less likely to contain desserts and sugar-sweetened beverages. These results indicate that the *a*DQI has construct validity and, therefore, can be used to objectively assess the healthfulness of food-related toy sets. Four caveats to use of the DQI for this purpose include (1) the DQI categorizes all vegetables the same such that starchy vegetables are indistinguishable; (2) proteins are not separated out as “healthy” (grilled) versus “less healthy” (fried)”; (3) the index does not include any measure of fats; and (4) since white wheat has become more prevalent on the market since 2002 to increase consumption of whole grains,^15^ we were not able to ascertain if grains in toy sets were representing refined grains or white wheat.

An additional limitation is that assessment of nutritional content of foods in the toy sets relied on appearance and not from actual food labels, and thus foods may have been misclassified. There was also no assessment of the popularity of the toy sets, and toy sets were not observed while being used by children, so there is lack of clarity about ways children use these toy sets.

Results of this study suggest potential ways toy manufacturers can improve healthfulness of food-related toy sets, which include switching from refined to whole grains, providing healthier protein choices, including more fruits and fewer desserts, and striving to ensure food-related toy sets are more representative of official dietary recommendations. Parents, child care facilities, and other agencies serving children may want to provide healthier food-related toy sets for pretend play. Finally, as there are no studies examining the association between children's dietary behaviors and food-related toy sets and pretend play, it will be important for future research to examine the effect of exposure to food-related toy sets on children's dietary behaviors.

## Supplementary Material

Supplemental data

Supplemental data

Supplemental data
