# Supplementary material for: Adaptation of a Modified Diet Quality Index to Quantify Healthfulness of Food-Related Toy Sets
Source: Child Obes. 2022 Aug 29;18(6):433–6. doi: 10.1089/chi.2021.0273 (PMC9492788; doi:10.1089/chi.2021.0273)
Supplement: Supplemental data [file Suppl_TableS1.docx]

Supplementary Table 1. Example of how independent variables were recorded for each toy set.

| **Toy Name** | FUNERICA Cutting Toy Play Food Set |
| --- | --- |
| **Retail Source** | Amazon |
| **Manufacturer** | FUNERICA |
| **Date reviewed** | 6/14/2020 |
| **Initials reviewer** | RW |
| **Price** | $24.61 |
| **# Total Items** | 43 |
| **# Food groups** | 5 |
| **# Condiment items** | 0 |
| **# Unidentifiable items** | 0 |
| **# Non-food items (including utensils)** | 7 |
| **# Cooking items** | 7 |
| **list cooking/food prep utensils** | cutting board, skillet, grill pan, spatula, knife, plates (2) |
| **# Scored items** | 36 |
