# Supplementary figures and images for: Adaptation of a Modified Diet Quality Index to Quantify Healthfulness of Food-Related Toy Sets
Source: Child Obes. 2022 Aug 29;18(6):433–6. doi: 10.1089/chi.2021.0273 (PMC9492788; doi:10.1089/chi.2021.0273)

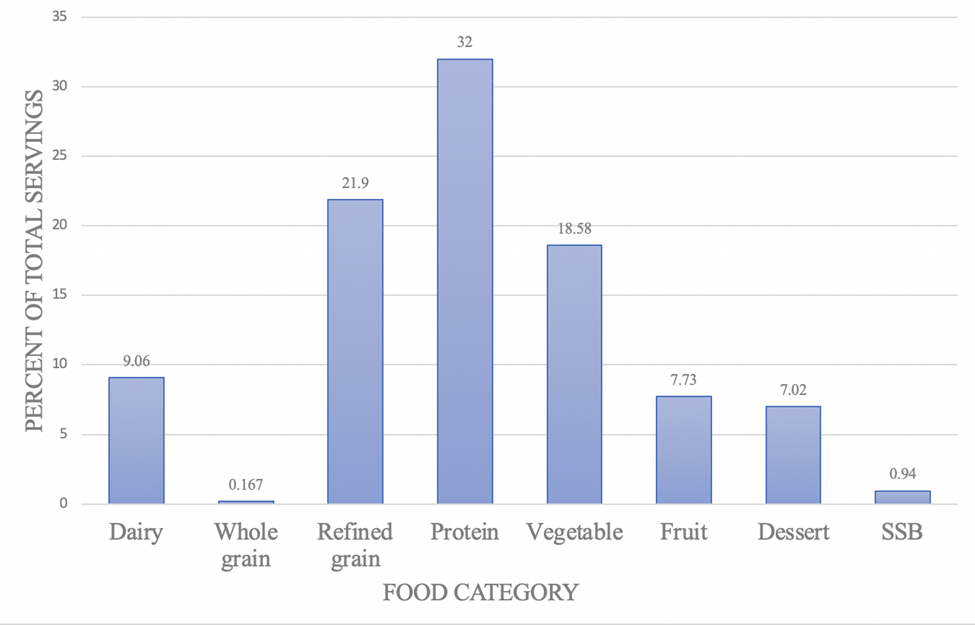
Supplementary Figure 1:

Supplement: Supplemental data [file Suppl_FigureS1.docx]
