# Supplementary material for: Adaptation of a Modified Diet Quality Index to Quantify Healthfulness of Food-Related Toy Sets
Source: Child Obes. 2022 Aug 29;18(6):433–6. doi: 10.1089/chi.2021.0273 (PMC9492788; doi:10.1089/chi.2021.0273)
Supplement: Supplemental data [file Suppl_FigureS2.docx]

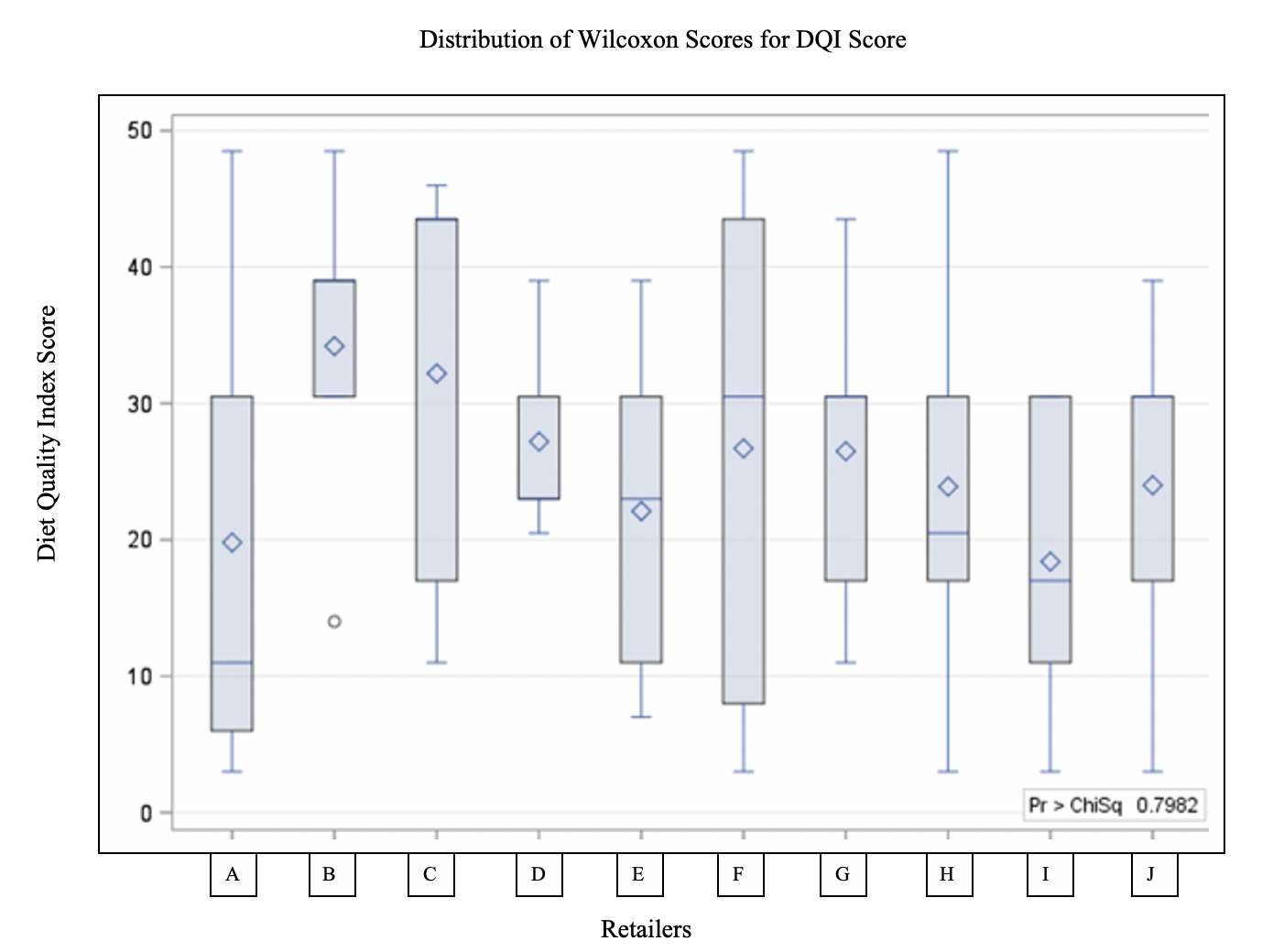


Supplementary Figure 2: Distribution of Wilcoxon scores for the DQI scores for each Retailer. A= Walmart, B = Target, C = Learning Resources, D = Sears, E = Bed Bath & Beyond, F = Amazon, G = Pottery Barn, H = Kohl’s, I = Macy’s, J = Best Buy.
